# P-683. Estimation of RSV-attributable cardiovascular and respiratory hospitalizations in adults in Germany between 2015–2019

**DOI:** 10.1093/ofid/ofae631.879

**Published:** 2025-01-29

**Authors:** Caroline Beese, Caihua Liang, Lea Johanna Bayer, Bennet Huebbe, Pimnara Peerawaranun, Robin Bruyndonckx, Aleksandra Polkowska-Kramek, Maribel Casas, Thao Mai Phuong Tran, Worku Biyadgie Ewnetu, Christof Von Eiff, Bradford D Gessner, Elizabeth Begier, Gernot Rohde

**Affiliations:** Pfizer Pharma GmbH, Berlin, Berlin, Germany; Pfizer Inc, New York, New York; Pfizer Pharma GmbH, Berlin, Berlin, Germany; n.a., Berlin, Berlin, Germany; P95, Chatuchak, Krung Thep, Thailand; P95, Chatuchak, Krung Thep, Thailand; P95, Chatuchak, Krung Thep, Thailand; P95, Chatuchak, Krung Thep, Thailand; P95, Chatuchak, Krung Thep, Thailand; P95, Chatuchak, Krung Thep, Thailand; Pfizer Pharma gmbH, Berlin, Berlin, Germany; Pfizer Biopharma Group, Collegeville, Pennsylvania; Pfizer Vaccines, Dublin, Dublin, Ireland; Goethe University Frankfurt, Frankfurt am Main, Hessen, Germany

## Abstract

**Background:**

RSV can cause severe outcomes among adults, especially those with underlying comorbidities. However, RSV incidence in adults is frequently underestimated due to non-specific symptomatology, limited standard-of-care testing and lower test sensitivity compared to infants. To address these gaps, we conducted a retrospective study to estimate RSV-attributable incidence of hospitalizations among adults in Germany between 2015–2019.

**Methods:**

Information on hospitalizations was gathered from a Statutory Health Insurance (SHI) database. A quasi-Poisson regression model was fitted to estimate RSV-attributable incidence rate (IR) of four specific cardiovascular hospitalizations (arrythmia, ischemic heart diseases, chronic heart failure exacerbations, cerebrovascular diseases) and four specific respiratory hospitalizations (influenza/pneumonia, bronchitis/bronchiolitis, chronic lower respiratory diseases, upper respiratory diseases) accounting for within-season fluctuations and virus activity stratified by age groups (18–44, 45–59, ≥60, 60–74, ≥75 years).

**Results:**

RSV-attributable IRs of hospitalizations were generally increasing with age. Among cardiovascular hospitalizations in adults aged ≥60 years, arrythmia and ischemic heart diseases accounted for the highest incidence, followed by chronic heart failure exacerbation, with annual IR ranges of 157–260, 133–214, and 105–169 per 100,000 person-years, respectively. Among respiratory hospitalizations, highest RSV-attributable incidence in adults aged ≥60 years were for chronic lower respiratory diseases and bronchitis/bronchiolitis, with annual IR ranges of 103–168 and 77­­–122 per 100,000 person-years, respectively.

**Conclusion:**

RSV infection is a well-established trigger for chronic heart failure exacerbations, but in this study RSV-related arrythmia and ischemic events were detected to have a higher incidence. RSV causes a considerable burden of both respiratory and cardiovascular hospitalizations in adults in Germany, similar to other respiratory viruses (e.g., influenza and SARS-CoV-2). This highlights the need to implement effective prevention strategies, especially for the older adults.

**Disclosures:**

**Caroline Beese, n/a**, Pfizer Pharma GmbH: Employee **Caihua Liang, MD, PhD**, Pfizer: Stocks/Bonds (Private Company) **Lea Johanna Bayer, n/a**, Pfizer Pharma: Employee **Bennet Huebbe, n/a**, Pfizer Pharma GmbH: Ex-employee **Aleksandra Polkowska-Kramek, n/a**, Pfizer: employee of P95 which received funding from Pfizer to conduct this study **Maribel Casas, PhD**, Pfizer: Advisor/Consultant **Christof Von Eiff, n/a**, Pfizer Pharma: I am working as Sen. Medical Direktor for Vaccines at Pfizer Pharma GmbH, Berlin, , Germany and in that position I also get stocks. **Bradford D. Gessner, M.D., M.P.H.**, Pfizer: Employee|Pfizer: Stocks/Bonds (Public Company) **Elizabeth Begier, MD, M.P.H.**, Pfizer Vaccines: Employee|Pfizer Vaccines: Stocks/Bonds (Private Company) **Gernot Rohde, MD**, Astra Zeneca: Advisor/Consultant|Astra Zeneca: Honoraria|Berlin Chemie: Honoraria|Boehringer Ingelheim: Honoraria|GSK: Advisor/Consultant|GSK: Honoraria|Insmed: Advisor/Consultant|Insmed: Honoraria|Pfizer: Honoraria|sanofi: Advisor/Consultant|sanofi: Honoraria

